# Whole-Brain Functional Connectivity Identification of Functional Dyspepsia

**DOI:** 10.1371/journal.pone.0065870

**Published:** 2013-06-17

**Authors:** Jiaofen Nan, Jixin Liu, Guoying Li, Shiwei Xiong, Xuemei Yan, Qing Yin, Fang Zeng, Karen M. von Deneen, Fanrong Liang, Qiyong Gong, Wei Qin, Jie Tian

**Affiliations:** 1 School of Life Sciences and Technology, Xidian University, Xi’an, Peoples R China; 2 The 3rd Teaching Hospital, Chengdu University of Traditional Chinese Medicine, Chengdu, China; 3 Department of Radiology, The Center for Medical Imaging, Huaxi MR Research Center (HMRRC), West China Hospital of Sichuan University, Chengdu, Sichuan, China; Institute of Psychology, Chinese Academy of Sciences, China

## Abstract

Recent neuroimaging studies have shown local brain aberrations in functional dyspepsia (FD) patients, yet little attention has been paid to the whole-brain resting-state functional network abnormalities. The purpose of this study was to investigate whether FD disrupts the patterns of whole-brain networks and the abnormal functional connectivity could reflect the severity of the disease. The dysfunctional interactions between brain regions at rest were investigated in FD patients as compared with 40 age- and gender- matched healthy controls. Multivariate pattern analysis was used to evaluate the discriminative power of our results for classifying patients from controls. In our findings, the abnormal brain functional connections were mainly situated within or across the limbic/paralimbic system, the prefrontal cortex, the tempo-parietal areas and the visual cortex. About 96% of the subjects among the original dataset were correctly classified by a leave one-out cross-validation approach, and 88% accuracy was also validated in a replication dataset. The classification features were significantly associated with the patients’ dyspepsia symptoms, the self-rating depression scale and self-rating anxiety scale, but it was not correlated with duration of FD patients (*p*>0.05). Our results may indicate the effectiveness of the altered brain functional connections reflecting the disease pathophysiology underling FD. These dysfunctional connections may be the epiphenomena or causative agents of FD, which may be affected by clinical severity and its related emotional dimension of the disease rather than the clinical course.

## Introduction

Functional dyspepsia (FD), as a complex and symptom-based disorder, was diagnosed based on patients’ subjective reports and their clinical manifestation. Several theories have been put forward to elucidate discomfort symptoms in FD, such as visceral hypersensitivity [1]–[6], the delay of gastric emptying [1], [6], [7], abnormal gastrointestinal motility [8], impaired gastric accommodation [6], [9], *Helicobacter pylori* infection [2], [6] and psychosocial factors [10]. Although self-reported symptoms and basic theories could provide useful clinical information, these studies only reflected a branch of the pathophysiology of FD. In recent years, neuroimaging studies provided unique insights in investigating brain dysfunction in functional gastrointestinal disease, which revealed the disease pathology based on the bidirectional interaction between the brain and gut [11]–[13]. The brain-gut interaction evaluated the input from the periphery via the visceral afferent system in the brain, which linked the cause of the disease to the central nervous system. To date, the neural mechanism that underlies the discomfort sensation in FD patients is not well understood.

Recently, several PET studies of FD patients have described functional abnormalities in brain regions associated with gastric hypersensitivity during different states, such as gastric distention with different thresholds, sham gastric distention and no distention [14]–[17]. Our previous study examined the levels of glycometabolism during resting state in FD patients as compared with healthy controls (HC), finding higher levels of glycometabolism in multiple cortices including the prefrontal cortex (PFC), the temporal cortex and the limbic system [17]. These results may advance the understanding of the neural mechanism that underlies the brain-gut communication in FD patients. However, the above studies illustrated the pathomechanism of FD by detecting aberrant signals of isolated brain regions in patients, which may simplify the pathological processes. Neuroimaging research pointed out that the physiological effects of brain injury do not arise from the breakdown of one or more isolated regions, but rather from the dysfunction of distributed brain networks [18]–[23]. Furthermore, it is necessary to develop a neuroimaging-based biomarker to perform individualized diagnosis of FD. Given the above problems, a multivariate pattern analysis (MVPA) classifier was used to discern the whole-brain functional connectivity patterns and evaluate the power of altered functional connections for categorizing FD patients from controls.

To extract sufficient information for classification, MVPA applied a sophisticated pattern recognition method to the intricate patterns termed as features. By combining an objective neuroimaging analysis and self-reported measurements, the extracted features may be more informative for classification, which could reflect alterations of the brain functions on one hand and patients’ clinical manifestations on the other hand. As a clinical instrument, the Nepean dyspepsia index (NDI) is designed especially for FD [24]. It has undergone validated translation from Australian English [24] to American English [25], Malay [26] and Chinese [27], which demonstrated that NDI has been considered as a widely accepted clinical outcome measuring FD. Some studies on FD have also proven its effectiveness by evaluating the association between the individual NDI score and individual symptom parameters [28], [29] and discriminating dyspepsia patients from controls [30]. Our prior study exhibited the correlation between the signals of brain regions with increased glycometabolism and NDI scores for dyspepsia patients, indicating that NDI may be helpful for discerning the changes in brain activity within FD patients [17]. Here, we hypothesize that MVPA could capture the functional connections for distinguishing FD patients from controls by extracting brain patterns related to NDI scores.

In this study, we explored the whole-brain patterns of resting-state functional connectivity in FD patients with abdominal distention using the MVPA approach. The aim of this study was to develop a method to discern functional connections that could make a distinction between dyspepsia sufferers and controls. We determined altered functional connections by estimating between-group differences of the Pearson correlation coefficients for each pair of brain regions, and selected those that were correlated with NDI scores as features for classification. Then, we investigated whether or not the altered functional connections could reflect severity and clinical features of FD. By converting the dysfunctional connections of each patient into a connectivity severity index (CSI), we determined the relationship between the index and patients’ clinical properties such as the NDI symptom score, self-rating depression scale (SDS), self-rating anxiety scale (SAS) and disease duration.

## Materials and Methods

### 2.1 Ethical Statement

The study process and protocols were approved by the West China Hospital Subcommittee on Human Studies. All subjects signed written informed consent protocols.

### 2.2 Subjects

Fifty FD patients (25 females) were recruited in the study. All were 20–30 years old and right-handed. The diagnosis of FD was documented by postprandial distress syndrome in the Rome III criteria [31], [32]. The patients with epigastric pain as the major clinical manifestation were excluded to avoid inclusion of irritable bowel syndrome. They underwent detailed history taking, gastrointestinal endoscopy, abdominal ultrasonography and physical examination including blood, urine, stool, electrocardiogram, hepatic function and renal function. The subjects with a history of drug abuse, head trauma with loss of consciousness, psychiatric or neurological disorders, cardiovascular, respiratory, hepatic or renal illnesses, or other serious co-morbidities were excluded, as were pregnant or lactating women. None of the subjects suffered from gastric atrophy, ulcers or erosive gastroduodenal lesions, esophagitis or cholecystitis. In addition, fifty gender- and age- matched HC (27 females) were enrolled in the experiment. There was no evidence of chronic or acute illness, or gastrointestinal symptoms via personal history, physical examination and the NDI questionnaire.

One female HC was discarded for falling asleep during the scan. Another female HC and one male patient were removed for excessive head motions (>1.5 mm translation and/or >1.5°). The remaining 49 FD patients and 48 HC were eligible for this study. The available samples were divided into two groups: original samples and replication samples. We selected 80 age- and sex-matched subjects (40 patients and 40 controls) as the original samples, and the remaining 17 subjects (9 patients and 8 controls) were used as the replication samples. The demographics and clinical assessments are revealed in Table 1.

**Table 1:** Demographics and clinical characteristics of the subjects.

	HC	FD patients	*p*-value
**Original sample**			
Age (years)	22.925±1.5256	22.45±1.8529	>0.1
Gender (female/male)	20/20	20/20	>0.1
Height (cm)	169.75±12.75	169.9±13.08	>0.1
Weight (kg)	62.13±14.76	60.79±13.35	>0.1
Education (years)	15.98±0.97	16±1.09	>0.1
Duration (months)	–	37.8250±28.2197	–
NDI-QoL	87.4057±5.4378	76.6503±10.1738	<10^−7^
NDI Symptom Score	1.2250±1.9012	47.2±15.8668	<10^−29^
SDS	33.9250±5.7708	44.3750±9.9799	<10^−6^
SAS	32.3±5.8713	42.6250±7.1712	<10^−9^
**Replication sample**			
Age (years)	22.1250±1.126	23±1.4142	>0.1
Gender (female/male)	5/3	5/4	>0.1
Height (cm)	168.38±10.48	168.44±9.9	>0.1
Weight (kg)	61.5±10.76	60.2±9.09	>0.1
Education (years)	15.75±0.71	15.9±1.05	>0.1
Duration (months)	–	28.5556±19.4622	–
NDI-QoL	89.7285±3.3859	81.9349±9.3938	<0.05
NDI Symptom Score	3±3.7417	43.3333±11.5326	<10^−6^
SDS	34.3750±1.9955	41.6667±12.2793	>0.05
SAS	32±5.5291	38.4722±10.3603	>0.05

HC, healthy controls; FD, functional dyspepsia; NDI, Nepean dyspepsia index; QoL, quality of life; SDS, self-rating depression scale; SAS, self-rating anxiety scale.

### 2.3 NDI Questionnaire

All subjects completed the NDI questionnaire [24], which consisted of two distinct measures: a quality of life scale and a symptom checklist. Simplified NDI quality of life scale (NDI-QoL) [29] assessed the impact of the illness on 17 key areas of life (25 items) with each item being graded on a scale from 0–4 (0, not at all or not applicable; 1, a little; 2, moderately; 3, quite a lot; and 4, extremely ). The NDI symptom checklist focuses on 15 upper gastrointestinal symptoms. Each symptom was measured based on the sum of the scores from the frequency (0–4, ranging from “not at all” to “daily”), severity (0–5, ranging from “not at all” to “very severe”), and bothersomeness (0–4, ranging from “not at all” to “extremely bothersome”).

### 2.4 Psychological Assessments

All subjects were asked to complete SDS [33] and SAS [34], ranging from “ a little of the time ” to “ most of the time” for the anxiety/depression they felt.

### 2.5 Data Acquisition

Before the experiment, all subjects stopped all use of medications, coffee or other stimulants for 15 days. In the experiment, subjects were instructed to keep their eyes closed, relax and stay awake. The experiment was performed on a 3-T Siemens scanner (Allegra; Siemens Medical System) at the Huaxi MR Research Center, West China Hospital at Sichuan University, Chengdu, China. A standard birdcage head coil was used to minimize head motion. Functional MRI was acquired with a gradient echo EPI sequence with the following parameters: repetition time = 2000 ms, echo time = 30 ms, flip angle = 90°, field of view = 240*240 mm^2^, imaging matrix = 64×64, in-plane spatial resolution = 3.75*3.75 mm^2^, number of slices = 30, slice thickness = 5 mm, and no gaps. Each 6-min resting-state session generated 180 whole-brain volumes. After each scan, subjects were asked whether they were relaxed and awake during the scan to avoid the inclusion of a substandard session.

### 2.6 Data Pre-processing

Image preprocessing was performed in MATLAB using SPM5 software (http://www.fil.ion.ucl.ac.uk/spm/). For each scan, the first ten volumes were discarded to eliminate non-equilibrium effects of magnetization and allow subjects to get used to the scanning environment. The remaining 170 volumes were corrected by the following procedures. First, initial slice timing was performed to adjust the volumes for the interleaved slice acquisition. Next, the adjusted images were realigned to the first volume for head motion correction. We have confirmed that there was no significant difference between the patient group and healthy group for the 6 head movement parameters (|T|<1.3, *p*>0.05). Then, the realigned images were spatially transformed to the Montreal Neurological Institute space with manual examination of appropriate normalization in all subjects, followed by re-slicing to a cubic resolution, 3×3×3 mm^3^. Finally, the time series of each voxel from the images was linearly detrended and temporally filtered with a band-pass filter (0.01–0.08 Hz) to remove the effects of scanner drift and physiological noise. The filtered images were used for the subsequent analysis.

### 2.7 Functional Brain Network Construction

To define brain network nodes, we parcellated the brain into 1024 ROIs (512 for each hemisphere) using the AAL-atlas after removing cerebellar structures [35]. For every subject, the time series of each ROI was computed by averaging the time series over all voxels in that ROI. The white matter, cerebrospinal fluid, ventricles and rotation average signals were regressed to correct regional time series without global signal regression to avoid artifactual correlations [36]. The residual time series were used to form the functional brain network with the Pearson correlation coefficients (Fisher’s r-to-z transformation) between each pair of ROIs as the edges (functional connectivity). We measured the interregional synchronization with a 1024×1024 symmetrical coefficient matrix, resulting in 523776 dimensional features by linearizing the upper triangular part of the matrix except for the elements of the main diagonal.

### 2.8 Construction of Feature Sets

The matrix with 523776 dimensional features (edge or functional connectivity) resulted in the ‘curse of dimensionality’ problem for directly classifying dyspepsia patients from controls. More importantly, the majority of the features were redundant and useless, and only a small proportion of features could contribute to the disorder of the brain connectivity patterns, which would degrade the performance of classification and may not spot the disorganized brain functional networks. In this work, a leave-one-out cross validation (LOOCV) strategy was employed to estimate classification accuracy. During LOOCV, each sample was left out in turns while the remaining samples were used to train the classifier. All feature selection was always performed on the training set only, as elucidated in the following steps.

1. First, determine the new training set. Suppose that there are 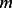 healthy samples and 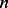 patient samples in the training set. Let 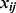 denote the feature 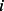 of the 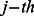 healthy subject, 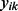 denote the feature 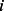 of the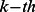 patient sample, and 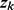 denote the NDI-QoL score of the 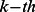 patient sample. 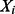 and 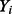 represent functional connectivity vectors of the feature 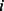 (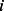 = 1,…,523776) for the healthy group and patient group respectively as follows:

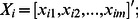
(1)

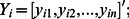
(2)

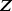
 represents the NDI-QoL vector for the patient group and is shown as:
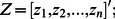
(3)
2. A *p*-value was calculated by a *t*-test comparing 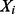 and 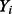, expressed as 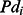. It represents the significance of real differences across two groups for the 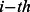 feature.
3. The features in 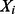 and 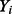 were randomly permutated 1000 times, and a *t*-test comparing 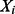 and 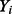 was repeated after each permutation, which generated 1000 *p*-values constituting an empirical estimate of the null distribution.
4. If 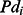 did not reach level 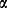 for significance of the distribution, then the 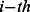 feature was excluded.
5. A *p*-value was obtained by computing the correlation of 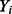 and 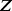, expressed as 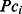. It represents the significance of the correlation between the functional connectivity vector of the feature 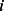 and the NDI-QoL vector for the patient group.
6. If 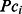 did not satisfy the significance of the correlation, then the 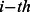 feature was eliminated.
7. The functional connections that were not removed constituted a new feature subset. The 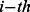 functional connectivity was retained in the feature subset only if 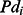 satisfied level 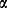 for significance and 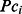 satisfied a significant correlation (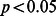). In the study, we set the range of 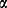 (0.05∼0.00001) to explore the optimal features for classification.
8. If each sample was left out once, the process should be terminated, otherwise, go to step1.

Since training samples were slightly different from iteration to iteration of the LOOCV, this process could result in N (the iterations of LOOCV) different feature subsets. Most predecessors selected the consensus features included in each iteration of the LOOCV (100% overlap) to finish their subsequent studies such as the discriminative power analysis and prediction [18], [20], [37]. However, some features provide effective information but are discarded from the consensus feature subset, which might be caused by only a few abnormal samples. Hence, setting an overlap threshold 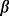 may be necessary to determine a stable feature subset, which could accurately classify patients vs. controls. The threshold should satisfy the criterion that the elements (features) in the stable feature subset were included in the overwhelming majority of the above N subsets.

Let *M* represent the occurrence number of one feature (functional connectivity) in *N* feature subsets, and then 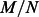 expresses the recurrent rate of the feature. If 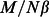, the feature would belong to the stable feature subset. Here, we set the range of 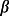 (100%, 95%, 90% and 85%) to explore the optimal feature subset for classification.

### 2.9 Performance Evaluation

The performance of classification was estimated by the sensitivity, specificity and accuracy. The sensitivity is the proportion of patients labeled accurately, while the specificity is the proportion of HC labeled correctly. Accuracy is the proportion of all samples labeled accurately. In addition, the statistical significance of accuracy was assessed using permutation tests [38]. The labels of the samples were randomly shuffled 1,000 times forming a null distribution of accuracy. If the real-label accuracy exceeded the 95% confidence interval for the null distribution, we rejected the null hypothesis that the classifier could not learn a real connection between the feature attributes and the class labels.

We used support vector machines to solve the classification and prediction problems. Computations related to the support vector machine were implemented using the LIBSVM Toolbox (www.csie.ntu.edu.tw/~cjlin/libsvm).

## Results

### 3.1 Clinical Variables

No significant group differences were observed in the demographics including age, gender, weight, height and education (*p*>0.05). The FD patients had significantly lower NDI-QoL scores (77.6±10.2 vs. 87.8±5.2, *p*<0.001) and higher NDI symptom scores (46.5±15.1 vs. 1.5±2.4, *p*<0.001) compared to controls.

The SDS scores and SAS scores were significantly higher in FD patients compared with controls (SDS, 43.9±10.4 vs. 34±5.3, *p*<0.001; SAS, 41.9±7.9 vs. 32.3±5.8, *p*<0.001). Fifteen of the FD patients showed mild depression (SDS>50), while none of the controls went beyond the normal range. Six of the FD patients but none of the controls showed mild anxiety (SAS>50).

As can be seen from Table 2, the NDI-QoL was strongly related only to NDI symptom score (*p*<0.05). There were significant correlations among the NDI symptom score, SDS and SAS (*p*<0.05). Nevertheless, no clinical variables showed significant correlations with the duration (*p*>0.05).

**Table 2:** Correlation among the clinical measures.

p-value	NDI-QoL	NDI symptom score	SDS	SAS	Duration
**NDI-QoL**	0	1.31e^−10^	0.23	0.27	0.97
**NDI symptom score**	1.31e^−10^	0	0.04	0.04	0.34
**SDS**	0.23	0.04	0	5.47e^−11^	0.77
**SAS**	0.27	0.04	5.47e^−11^	0	0.98
**Duration**	0.97	0.34	0.77	0.98	0

*P*-values less than 0.05 represent a significant correlation between the two groups. NDI, Nepean dyspepsia index; QoL, quality of life; SDS, self-rating depression scale; SAS, self-rating anxiety scale.

### 3.2 Classification and Prediction

**Figure 1:**
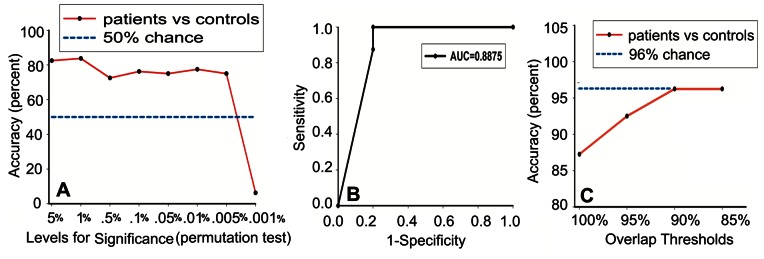
Whole-brain functional connectivity discriminates between functional dyspepsia patients and healthy controls within the original samples. (A) Classification accuracy of patients vs. controls as a function of the significance levels for the permutation test (0.05∼0.00001). Maximum accuracy (83.75%) was achieved at the level of 0.01. (B) Receiver operating characteristic curve based on the level of 0.01. (C) Classification accuracy with the change of the overlap thresholds (100%∼85%) at the significance level of 0.01. AUC, area under receiver operating characteristic curve.

Different levels 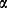 in the processing of feature construction represented different statistical significances, generating different feature sets. Although a high statistical power could reduce noise in the statistical analysis, it may blur out fine-grained feature patterns that may be able to distinguish between dyspepsia patients and controls. To attain a trade-off, a LOOCV classification accuracy for 40 FD patients and 40 controls was calculated over the significance levels for the permutation test ranging from 0.05 to 0.00001(Fig. 1 A). The maximum accuracy was found at 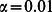, and the classifier correctly identified 32/40 HC (80%) and 35/40 FD patients (87.5%), with a total accuracy of 67/80 (83.75%). At 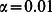, 80 different feature sets were obtained and the ROC curve was also calculated (Fig. 1 B).

The LOOCV approach assessed a reproducible result; however, the best classification may not be adequate due to the presence of sample-sensitive features from an outlier. In order to exclude the contribution of an outlier, different overlap thresholds (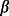 = 100%, 95%, 90% and 85%) were set to eliminate the sample-sensitive features, so that the stable feature subsets could be obtained. We then repeated the LOOCV classification within the original samples. The leave-one-out approach accuracy for 40 patients and 40 controls reached 0.88 (70/80), 0.92 (74/80), 0.96 (77/80) and 0.96 (77/80) when 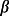 equaled 100%, 95%, 90% and 85% respectively (Fig. 1 C).

To further validate the discriminative power of the feature set (dysregulated functional connections), we tested the feature subsets for classifying the replication samples, including 9 FD patients and 8 HC. The results achieved peak accuracy of 0.88 (15/17) at 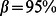
(7/9 patients and 8/8 controls) and 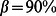 (8/9 patients and 7/8 controls), as revealed in Table 3. Moreover, the permutation test estimating the statistical significance of accuracy (*p*<0.05) indicated that the classifier could learn the dysfunctional FD-related connection and differentiate FD patients from healthy individuals with relatively high sensitivity and specificity.

**Table 3:** Classification performance of functional connections within the replication samples at different overlap thresholds.

Overlap thresholds	Number of features	Sensitivity	Specificity	Accuracy
**100%**	60	0.7778	0.875	0.8235
**95%**	67	0.7778	1	0.8824
**90%**	78	0.8889	0.8750	0.8824
**85%**	99	0.8889	0.75	0.8235

**Figure 2:**
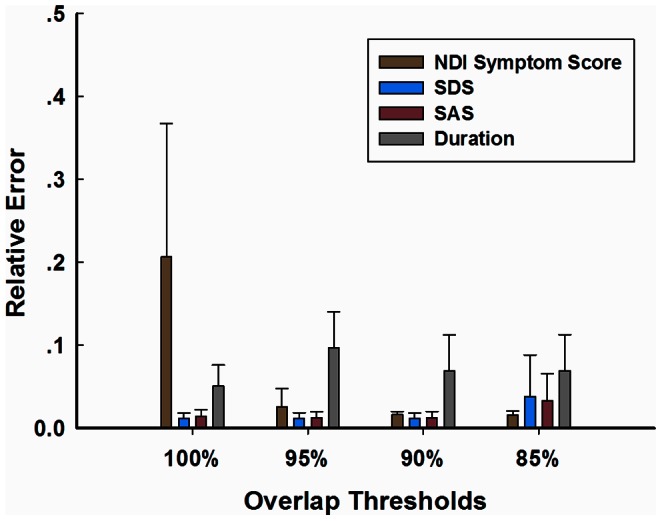
Relative errors of clinical variables showing predictive power of abnormal functional connections at different overlap thresholds (100%, 95%, 90% and 85%). The duration was poorly predicted at all thresholds and the best prediction of other variables was achieved at the threshold of 90%. NDI, Nepean dyspepsia index; SDS, self-rating depression scale; SAS, self-rating anxiety scale.

In addition, we predicted patients’ NDI symptom scores, SDS, SAS and disease duration using features at different overlap thresholds. The main purpose was to determine the optimal threshold by comparing the relatively predicted errors (|predicted value-actual value|/actual value). Overall, the prediction results showed the lowest errors at 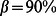 in comparison to other thresholds (Fig. 2). At 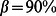, the NDI symptom score, SDS and SAS were predicted with relatively low errors, but duration was displayed with poor prediction (Fig. 2). In the results, the prediction for the patients’ disease duration exhibited high error at all thresholds.

### 3.3 Abnormal Connectivity in FD Patients

**Figure 3:**
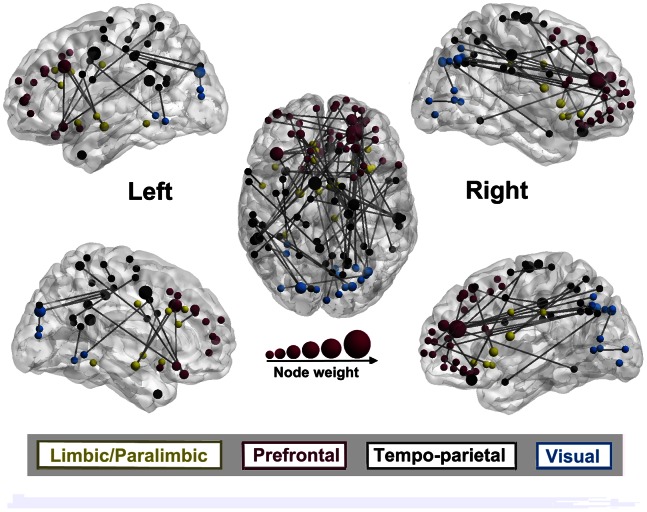
Abnormal functional network based on significance level 0.01 and overlap threshold of 90%. The nodal weights were scaled by the frequency of occurrences in aberrant connections. The biggest region was observed in the right dorsolateral part of the frontal superior gyrus (BA9).

The retained features at 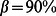 captured the altered brain functional connections in FD patients (Table S1). These alterations represented the impairment of neurocircuitry in dyspepsia patients, which was located within or across the limbic/paralimbic system, PFC, tempo-parietal regions and visual cortex. The abnormal brain network including ROIs and functional connections was revealed on a surface rendering of the human brain atlas using the BrainNet Viewer. As can be seen in Fig. 3, we visualized altered ROIs in terms of main cortical sites, each site corresponding to a specific structure in the human brain. The weight of each node varied with its frequency of occurrences in aberrant connections and indicated the contribution of each ROI to central abnormalities in FD patients (Fig. 3). The biggest ROI with the best classification power was in the dorsolateral part of the right superior frontal cortex (Talairach coordinate: 32, 35, 31; BA9; dlPFC).

In this study, we also assessed the relationships between the FD patients’ abnormal connectivity intensity and NDI-QoL across all subjects while controlling for age. The results showed that 65% of the aberrant connections exhibited negative correlations with the NDI-QoL. We also estimated the connectivity length by the Euclidean distance between the centroids of the respective ROIs in millimeters. The results uncovered that negatively correlated connections (mean length: 80.3 mm, standard deviation: 31.2 mm) were significantly longer (*t* = 2.097, *p*<0.05) than positively correlated connections (mean length: 65.9 mm, standard deviation: 23.4 mm).

**Figure 4:**
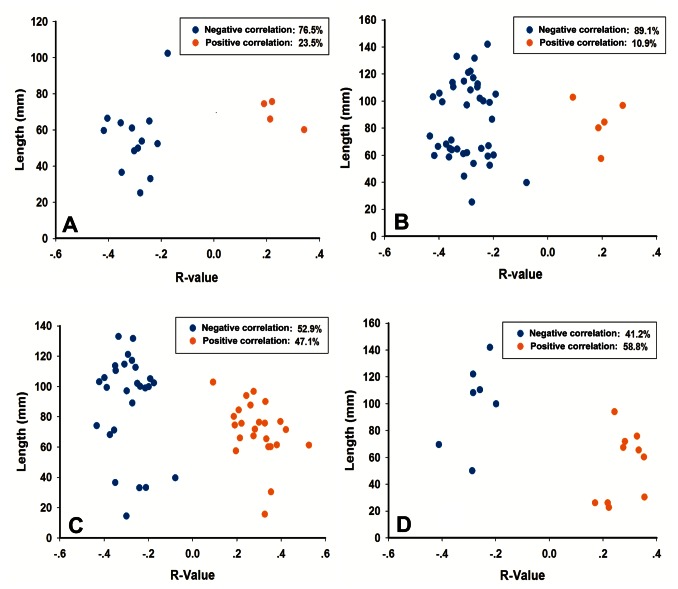
Characteristic patterns of abnormal functional connections within or across each cortical structure including (A) limbic/paralimbic system, (B) prefrontal cortex, (C) tempo-parietal areas and (D) visual cortex. Dots represent abnormal connections in FD patients. R-value is the coefficient of correlation between functional connectivity and quality of life score, and the length is the Euclidean distance.

As shown in Fig. 4, we observed that connections to the limbic/paralimbic structures and the PFC were predominantly negatively correlated with the NDI-QoL (the limbic/paralimbic system: 76.5%, the PFC: 89.1%). Focusing on the connections negatively associated with the NDI-QoL, those linked to the limbic/paralimbic system were shorter in length, while connections to the PFC had a wide distribution in length. The proportion of functional connections with negative correlations was not significantly different from those with positive correlations in the other two cortices. These findings may manifest excitatory and inhibitory pathways in FD patients’ brains.

### 3.4 Correlation between Clinical Measurements and Abnormal Connectivity

**Figure 5:**
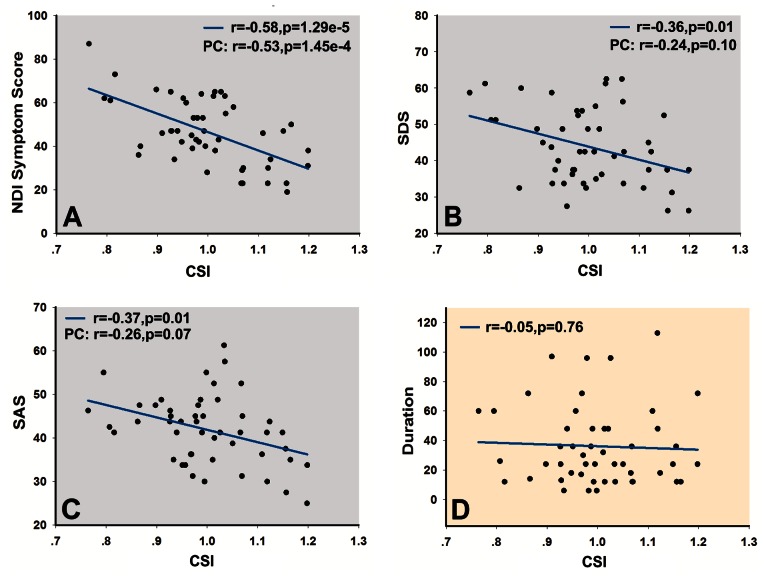
Relationship between connectivity severity index (CSI) and clinical measurements including (A) NDI symptom score, (B) SDS, (C) SAS, and (D) duration. The partial correlation (PC) between CSI and NDI symptom score persisted while controlling for the SDS and SAS, but the correlation between SAS/SDS and CSI disappeared with the NDI symptom score as a covariate. No significant association was found between the CSI and duration. NDI, Nepean dyspepsia index; SDS, self-rating depression scale; SAS, self-rating anxiety scale; r, correlation coefficient.

To determine the extent to which the abnormal functional connections in FD patients reflected the symptoms of dyspepsia and harmful emotions (depression/anxiety), the predicted NDI-QoL for each patient using abnormal functional connections was converted to a connectivity severity index (CSI) by setting the mean predicted NDI-QoL equal to 1.0 [37]. Then, the CSI represented a multi-dimensional indicator of the subjects’ health status. The lower the CSI, the more serious the illness was. We computed the correlation between the CSI and clinical scores within all patients (Fig. 5), and it showed that the CSIs were significantly associated with the NDI symptom score (*r* = −0.58, *p* = 1.29e^−5^), SDS (*r* = −0.36, *p* = 0.01), and SAS (*r* = −0.37, *p* = 0.01), but were not correlated with the duration of FD patients (*r* = −0.05, *p* = 0.76). In addition, we carried out partial correlation analysis to clarify the relationship between the CSI and clinical measures. The significantly negative correlation between CSI and NDI symptom persisted but was reduced while controlling for SAS and SDS (r = −0.53, p = 1.45e^−4^). With the NDI symptom score as a covariate, correlations between SDS/SAS and CSI disappeared (SDS-CSI: r = −0.24, *p* = 0.1; SAS-CSI: r = −0.26, *p* = 0.07).

## Discussion

This study demonstrated that whole-brain resting-state functional connectivity captured abnormal patterns in FD patients by MVPA, which could distinguish FD patients from HC with high accuracy. The discriminative power of altered functional connections was also validated in an independent sample set (the replication group) with high accuracy for avoiding biased estimation. The identified abnormal connections were converted to the CSI for each patient and we probed the relationship between the CSI and clinical metrics. The results showed that the CSIs were correlated with the NDI symptom score, SDS and SAS, but were not associated with the duration. Moreover, the correlation between CSIs and SDS/SAS disappeared while controlling for NDI symptoms. Our findings may provide more evidence of the brain pattern changes in FD patients and improve the understanding of the pathophysiology of FD.

FD is characterized by the absence of organic disease and the presence of recurrent symptoms including postprandial epigastric pain, abdominal distension, early satiety, bloating, nausea, belching and other abdominal discomfort. Postprandial abdominal distension was the chief sensation of dyspepsia patients in this study. Physically, gastric distention may induce the change in extensive brain regions [39]–[41]. In our study, the abnormal functional connections for best classification and prediction were located within or across the limbic/paralimbic system, the PFC, tempo-parietal areas as well as the visual cortex. Our results pointed toward an abnormal mode of brain function in patients with FD.

Previous researchers reported the significant roles of the limbic-cortical areas in visceral studies. For example, the amygdala (AMYG) processed social signals for emotion, emotional conditioning, the consolidation of emotional memories [42], and interoceptive signals of fullness [39]; the insula integrated visceral sensation [43], emotive feeling [44], [45], and autonomic visceromotor responses [46]; the anterior cingulate cortex (ACC) participated in emotion modulation, cognitive operation and visceral perception [43], [47]; the parahippocampus engaged in emotional processes for regulating visceral responses [48]; and the thalamus was deemed as a major relay station between the periphery at the visceral level and the brain cortex [49]. In this study, we found that the abnormalities were located in the AMYG, insula, ACC, parahippocampus, putamen, middle cingulated cortex and thalamus, which were similar to but not completely consistent with previous reports. The findings showed that the communication between the brain and gut was involved in multiple areas of the limbic/paralimbic system, supporting these prior notions. We thought the pathways of the limbic/paralimbic regions to other structures were likely to integrate sensory-discriminative, cognitive and affective-motivational functions related to the discomfort symptoms from the gastrointestinal tract.

The PFC was recently considered to be involved in the “visceral sensory neuromatrix” [50], emotional responses [51], and cognitive modulations of pain [52]. In the current results, we found many dysregulated connections related to the PFC including the orbitofrontal cortex, dlPFC, ventrolateral prefrontal cortex, and so forth. An fMRI study showed that gastric fundic distension activated the fronto-limbic network [53]. This study indicated that the brain circuit of the PFC to the limbic regions may be relevant to gastric distension symptoms, which was consistent with our findings. We suspected that impaired connections to the PFC may disrupt the multiple functions of cognitive-affective processing [54], [55] and sensory information [43] within FD patients with abdominal distension. As a result, the ROI with the biggest discriminative power was observed in the right dlPFC. The area was not only frequently reported in abnormal descending modulation [56], [57] and emotion-related regulation [58], but also viewed as a cognitive region [59], [60], which was involved in attention to and anticipation of visceral sensation in the brain-gut interaction. Our results seemed to suggest that the dlPFC played a key role in the communication between the brain and gut.

Tempo-parietal areas are composed of widespread brain regions. In this study, we documented aberrations from tempo-parietal clusters within FD patients. Human neuroimaging studies reported the response to gastric distention within these cortices engaging in the central processing of fullness [14], [15], [39], [61], [62] in accordance with our observations. Although it remains controversial that these cortices mediated gastric sensation [41], [53], [63], the results suggest that the tempo-parietal areas may play a significant role in neuronal pathways from the gastrointestinal peripheral tracts to the cerebral cortex.

Compared with the HC resting network, functional connections showed disorders in the FD patients’ networks referring to the occipital cortex, lingual cortex, calcarine and fusiform cortex which were recognized as the visual cortex. By using 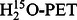, Van Oudenhove et al. reported abnormal activity in the occipital areas in FD [15]. Furthermore, several previous studies observed a significant correlation between the activation in the visual cortex and gastric distention [15], [41]. According to our findings, the dysregulated connections to the visual cortex in FD patients may be the result of aberrant brain activities related to visceral functions. Currently, few studies explained visual areas in connection with visceral activity and they speculated that this may be due to selective attention to gastric distention in FD patients. These explanations remain speculative and deserve further confirmation.

In the study, the negatively correlated connections had a predominant proportion, especially for the limbic/paralimbic system and the PFC, which are the most important structures in gut-associated brain areas. Focusing on the limbic/paralimbic system and PFC, we found that connections to the limbic/paralimbic system were shorter than connections to the PFC. Nearby functional regions are prone to belong to the same functional network, or play an interrelated role in the human brain. We suspected that the aberrations to the limbic/paralimbic regions were inclined to be intra-modular connections while the abnormalities in the PFC had both intra- and inter- modular connections. Although different roles are played in the regulation of gastrointestinal functions, these disorder connections (both intra- and inter-modular) may disrupt the network topology, indicating the change in efficiency and cost of the brain network.

For individuals with FD, the NDI symptom score, SDS, SAS and duration were important measurements since they corresponded to the symptoms of dyspepsia, passive affective states (depression or anxiety) and the course of the disease. The CSI we defined is a metric of multi-dimensional functional connectivity. The relationship of clinical measurements and CSI may manifest whether or not the abnormalities of brain functional connectivity were directly involved in the pathophysiology of FD.

The NDI symptom score is a useful measure for gastrointestinal symptoms of FD patients, and it directly reflects the severity in FD patients [24]. Thus, we inferred that the CSIs were strongly correlated with NDI symptom scores based on all patients. In our study, the association between the NDI symptom scores and CSIs indicated that the CSI satisfied the validation of the definition. SDS and SAS measure the behavioral manifestations of passive emotions with depression and anxiety respectively [33], [34]. In our results, after controlling for the NDI symptom scores, the loss of correlation between CSIs and SDS/SAS seemed to suggest that the observed functional connectivity abnormalities were driven by dyspepsia symptoms as opposed to psychological/psychiatric effects. However, a higher prevalence of depression/anxiety was found in FD patients than in HC [64], [65]. Moreover, many studies confirmed the correlation between depression/anxiety and FD, suggesting that emotional factors might influence the pathophysiology or symptoms directly in FD [10], [65], [66]. In the present study, SAS/SDS exhibited a significant correlation with the NDI symptom score. It indicated that the influences of emotional factors on FD might be reflected in the dyspepsia symptoms, which may lead to the loss of partial correlation between SDS/SAS and CSI (NDI symptoms as a covariate). Duration is usually an important parameter for a clinician. However, the CSI here was not related to the duration within patients. This signified that aberrant brain circuits may not reveal the course of FD or the duration may not be a good measurement for the severity of FD in accordance with the conclusion that the symptoms of functional gastrointestinal disorder were stable over time [67].

### Conclusion

In summary, our findings highlighted the remarkable brain functional connectivity alterations in FD patients at rest, which could be used to identify patient individuals from controls with high classification accuracy. The relationship between abnormal connections and clinical variables suggested that functional connectivity patterns may reflect the severity of the FD disease. Improved understanding of FD-related brain functional changes may provide novel insights into the pathophysiological mechanism of the disease. Our results suggest the potential utilization of brain functional connectivity as a disease biomarker.

## Supporting Information

Table S1 Abnormal functional connections of functional dyspepsia patients.
(DOC)
