# Supplementary material for: Whole-Brain Functional Connectivity Identification of Functional Dyspepsia
Source: PLoS One. 2013 Jun 17;8(6):e65870. doi: 10.1371/journal.pone.0065870 (PMC3684590; doi:10.1371/journal.pone.0065870)
Supplement: Table S1 — Abnormal functional connections of functional dyspepsia patients. (DOC) [file pone.0065870.s001.doc]

Table S1. Abnormal functional connections of functional dyspepsia patients.

| Abnormal connections | | | | | | | | | | |
| --- | --- | --- | --- | --- | --- | --- | --- | --- | --- | --- |
| Region 1 |  | Talairach Coordinate | | |  | Region 2 |  | Talairach Coordinate | | |
|  |  | x | y | z |  |  |  | x | y | z |
| Amygdala | L | -21 | -7 | -13 |  | Middle Frontal | L | -39 | 25 | 32 |
| Amygdala | L | -21 | -7 | -13 |  | Superior Frontal | L | -24 | 35 | 43 |
| Anterior Cingulate | L | -9 | 36 | 20 |  | Middle Frontal | L | -27 | 36 | 29 |
| Middle Cingulate | L | -6 | -7 | 34 |  | Inferior Frontal | L | -14 | 26 | -18 |
| Middle Cingulate | L | -9 | 19 | 27 |  | Putamen | R | 21 | 17 | -6 |
| Middle Cingulate | L | -9 | 33 | 29 |  | Precentral | R | 18 | -23 | 67 |
| Middle Cingulate | R | 3 | -1 | 33 |  | Superior Frontal | L | -6 | 60 | 25 |
| Middle Cingulate | R | 3 | -27 | 35 |  | Middle Occipital | R | 13 | -90 | 14 |
| Insula | L | -39 | 11 | -6 |  | Medial Frontal | R | 9 | 37 | -9 |
| Insula | L | -42 | 3 | -3 |  | Inferior Frontal | L | -60 | 12 | 26 |
| Insula | R | 36 | 14 | -6 |  | Superior Frontal | R | 9 | 62 | 11 |
| Insula | R | 35 | 12 | -3 |  | Rectus | R | 3 | 34 | -19 |
| Insula | R | 36 | 15 | -1 |  | Superior Frontal | R | 21 | 29 | 54 |
| Insula | R | 33 | 15 | -1 |  | Paracentral lobule | L | -3 | -40 | 54 |
| Thalamus | R | 6 | -5 | 11 |  | Superior Parietal | R | 21 | -61 | 58 |
| Thalamus | R | 6 | -5 | 11 |  | Superior Parietal | R | 30 | -52 | 58 |
| ParaHippocampal | L | -30 | -41 | -8 |  | Paracentral lobule | L | 6 | -29 | 61 |
| Inferior Frontal | R | 59 | 18 | 16 |  | Parietal | R | 3 | -62 | 34 |
| Inferior Frontal | L | -21 | 11 | -16 |  | Inferior Frontal | R | -15 | 28 | -17 |
| Middle Frontal | L | -37 | 60 | 10 |  | Superior Frontal | R | 21 | 57 | 28 |
| Middle Frontal | L | -37 | 60 | 10 |  | Superior Frontal | R | 32 | 35 | 31 |
| Middle Frontal | L | -39 | 25 | 32 |  | Medial Frontal | R | 3 | 34 | -14 |
| Middle Frontal | L | -39 | 25 | 32 |  | Rectus | R | 3 | 34 | -19 |
| Middle Frontal | L | -48 | 49 | -5 |  | Inferior Frontal | R | 39 | 31 | -12 |
| Middle Frontal | L | 42 | 46 | -12 |  | Middle Frontal | L | -39 | 25 | 32 |
| Middle Frontal | R | 33 | 55 | -8 |  | Superior Frontal | R | 21 | 43 | 45 |
| Middle Frontal | R | 36 | 55 | -3 |  | Rectus | R | 3 | 34 | -19 |
| Middle Frontal | R | 33 | 47 | 9 |  | Parietal | R | 6 | -61 | 41 |
| Superior Frontal | L | -17 | 24 | 53 |  | Precentral | R | 21 | -17 | 67 |
| Superior Frontal | L | -29 | 59 | 5 |  | Parietal | R | 15 | -68 | 34 |
| Superior Frontal | L | -21 | 45 | 23 |  | Lingual | R | 12 | -82 | -9 |
| Superior Frontal | L | -21 | 39 | 31 |  | Precentral | R | 21 | -17 | 67 |
| Superior Frontal | L | -21 | 39 | 31 |  | Precentral | R | 18 | -23 | 67 |
| Medial Frontal | R | 5 | 42 | -12 |  | Paracentral Lobule | R | -4 | -32 | 52 |
| Superior Frontal | R | 25 | 51 | 2 |  | Parietal | R | 6 | -61 | 41 |
| Superior Frontal | R | 24 | 62 | 13 |  | Middle Frontal | R | 30 | 20 | 54 |
| Superior Frontal | R | 21 | 29 | 54 |  | Inferior Parietal Lobule | R | -59 | -38 | 36 |
| Superior Frontal | R | 21 | 57 | 28 |  | Middle Occipital | R | 13 | -90 | 14 |
| Superior Frontal | R | 21 | 57 | 28 |  | Middle Frontal | R | 27 | 28 | -17 |
| Inferior Frontal | R | 48 | 16 | 24 |  | Parietal | L | -20 | -29 | 65 |
| Middle Frontal | L | 42 | 46 | -12 |  | Inferior Parietal | L | -49 | -32 | 36 |
| Middle Frontal | R | 39 | 37 | 34 |  | Middle Temporal | L | -50 | -60 | 22 |
| Medial Frontal | R | 6 | 62 | 19 |  | Postcentral | L | -33 | -32 | 61 |
| Inferior Frontal | L | -14 | 26 | -18 |  | Paracentral lobule | L | -3 | -40 | 54 |
| Superior Frontal | R | 24 | 64 | 0 |  | Precentral | R | 21 | -17 | 67 |
| Superior Frontal | R | 15 | 37 | 48 |  | SupraMarginal | L | -56 | -45 | 24 |
| Superior Frontal | R | 21 | 67 | 2 |  | SupraMarginal | L | -56 | -45 | 24 |
| Superior Frontal | R | 21 | 43 | 45 |  | SupraMarginal | L | -56 | -45 | 24 |
| Superior Frontal | R | 15 | 35 | 51 |  | Inferior Parietal | L | -49 | -32 | 36 |
| Superior Frontal | R | 24 | 12 | 57 |  | Middle Temporal | L | -36 | 7 | -38 |
| Superior Frontal | R | 21 | 12 | 57 |  | Middle Temporal | L | -36 | 7 | -38 |
| Superior Frontal | R | 32 | 35 | 31 |  | Superior Occipital | R | 33 | -80 | 32 |
| Superior Frontal | R | 32 | 35 | 31 |  | Superior Occipital | R | 42 | -82 | 22 |
| Superior Frontal | R | 32 | 35 | 31 |  | Superior Parietal | R | 12 | -67 | 53 |
| Superior Frontal | R | 32 | 35 | 31 |  | Superior Parietal | R | 36 | -53 | 52 |
| Superior Frontal | R | 32 | 35 | 31 |  | Inferior Temporal | R | 53 | -50 | -13 |
| Fusiform | L | -36 | -50 | -13 |  | Superior Temporal | L | -44 | -44 | 19 |
| Fusiform | L | -29 | -49 | -10 |  | Precentral | L | -36 | 8 | 36 |
| Lingual | R | 6 | -67 | 3 |  | Lingual | R | 1 | -88 | 2 |
| Middle Occipital | L | -36 | -84 | 18 |  | Middle Occipital | R | 24 | -95 | 14 |
| Middle Occipital | R | 29 | -95 | 17 |  | Temporal | R | 48 | -1 | -13 |
| Superior Occipital | L | -21 | -86 | 32 |  | Lingual | L | -6 | -87 | 5 |
| Superior Occipital | L | -21 | -86 | 32 |  | Inferior Parietal | L | -50 | -30 | 43 |
| Superior Occipital | L | -21 | -86 | 32 |  | Parietal | L | -57 | -21 | 41 |
| Cuneus | R | 12 | -73 | 16 |  | Superior Occipital | L | 15 | -83 | 35 |
| Middle Occipital | L | -15 | -86 | 35 |  | Postcentral | L | -53 | -24 | 48 |
| Middle Occipital | R | 34 | -87 | 18 |  | Inferior Parietal | L | -39 | -41 | 52 |
| Middle Occipital | R | 34 | -87 | 18 |  | Parietal | L | -57 | -21 | 41 |
| Precentral | L | -24 | -26 | 57 |  | Postcentral | L | -20 | -30 | 65 |
| Precuneus | L | -12 | -57 | 28 |  | Precentral | L | -36 | -6 | 56 |
| Precuneus | L | -6 | -49 | 61 |  | Precentral | R | 56 | 1 | 28 |
| Postcentral | L | -52 | -13 | 17 |  | Precuneus | L | -6 | -49 | 61 |
| Cuneus | R | 3 | -68 | 31 |  | Precuneus | R | 6 | -56 | 42 |
| Postcentral | R | 18 | -35 | 65 |  | Postcentral | R | -27 | -35 | 63 |
| Inferior Parietal | R | 53 | -30 | 44 |  | Superior Parietal | R | -25 | -48 | 60 |
| Postcentral | L | -5 | -42 | 64 |  | Inferior Parietal | R | 53 | -30 | 44 |
| Paracentral Lobule | L | -3 | -40 | 54 |  | Inferior Parietal | R | 53 | -30 | 44 |
| Paracentral Lobule | L | -3 | -40 | 54 |  | Inferior Parietal | R | 40 | -30 | 40 |

L: left hemisphere; R: right hemisphere.
